# Factors Associated with Growth and Weight Status in Indigenous Children Under 5 Years of Age in Northeastern Brazil

**DOI:** 10.1007/s10995-026-04274-z

**Published:** 2026-05-14

**Authors:** Ana Beatriz Sousa Luz dos Anjos, Valéria Clarisse de Oliveira, Tamara Rodrigues dos Santos, Haroldo da Silva Ferreira, Marly Augusto Cardoso, Bárbara Hatzlhoffer Lourenço

**Affiliations:** 1 Public Health Nutrition Program, School of Public Health, University of São Paulo, São Paulo, Brazil; 2 Public Health Program, School of Public Health, University of São Paulo, São Paulo, Brazil; 3 School of Nutrition, Federal University of Alagoas, Maceió, Brazil; 4 Department of Nutrition, School of Public Health, University of São Paulo, Avenida Doutor Arnaldo 715, São Paulo, SP 01246-904 Brazil; 5 Global Health and Tropical Medicine, GHTM, Associate Laboratory in Translation and Innovation Towards Global Health, LA-REAL, Institute of Hygiene and Tropical Medicine, IHMT, NOVA University of Lisbon, UNL, Lisbon, Portugal

**Keywords:** Nutritional status, Indigenous children, Child nutrition, Stunting, Overweight

## Abstract

**Objective:**

Indigenous children in Brazil experience persistent health and nutritional inequities, yet remain underrepresented in epidemiological research. This study investigated factors associated with the nutritional status of Indigenous children under 5 years in Northeastern Brazil.

**Methods:**

A cross-sectional household survey collected demographic, socioeconomic, maternal, perinatal, and anthropometric data of 329 children across 13 Indigenous communities in Alagoas, Brazil. Height-for-age (HAZ) and body mass index-for-age (BAZ) z-scores were calculated using the World Health Organization growth standards. Multiple linear regression models were used to estimate coefficients (β) and confidence intervals (95%CI) of factors associated with HAZ and BAZ.

**Results:**

Among 329 Indigenous children (52% boys, mean age [standard deviation, SD]: 2.4 [1.4] years), the mean HAZ was − 0.38 (SD: 1.09) and BAZ was 0.50 (SD: 1.10). Overall, 6.1% of children were stunted (HAZ <-2) and 9.5% were overweight (BAZ > + 2). HAZ was negatively associated with moderate or severe food insecurity (β: −0.37; 95%CI: −0.71, −0.03). Children living in masonry households (β: 0.71; 95%CI: 0.10, 1.32) and whose mothers were taller (β: 0.54; 95%CI: 0.24, 0.85) had higher HAZ. Regarding weight status, maternal underweight (β: −1.02; 95%CI: −2.00, −0.03) and cesarean section (β: 0.38; 95%CI: 0.14, 0.63) were significantly associated with BAZ.

**Conclusions for Practice:**

Monitoring the nutritional status of Indigenous children is important in the first years of life. Strengthening traditional practices within Indigenous territories is essential for improved nutritional outcomes.

## Significance

What is Already Known on This Subject?

What This Study Adds?

Childhood growth processes are critical to the health and well-being of populations. Vulnerable groups, including Indigenous populations, have been historically marginalized in epidemiological studies, and targeted research is needed.

This study provides a comprehensive understanding of the distribution of different forms of malnutrition among Indigenous children in Northeastern Brazil, with the coexistence of 6.1% of stunting and 9.5% of overweight. Shared social, environmental, and health factors should be considered to prevent suboptimal growth and higher weight status in the first 5 years of life, including food security, better socioeconomic, maternal health and prenatal conditions.

## Introduction

Child growth is a key determinant of population health and well-being, and is directly linked to the 2030 Sustainable Development Goals. Global estimates for 2022 showed that stunting affected 21.3% (148.1 million) children under 5 years of age, while 6.7% (approximately 37 million) had overweight (UNICEF, WHO, & World Bank, 2023). In Brazil, national trends from 2006 to 2019 showed a persistent prevalence of stunting (7%), alongside a marked increase in overweight (from 6% to 10.1%) (Castro et al. 2023a, 2023b).

Vulnerable population groups, such as Indigenous communities in Brazil, are more likely to be affected by malnutrition and related health inequities, including higher infant mortality and exposure to environmental and territorial crises. These communities have historically experienced marginalization, discrimination, and limited inclusion in epidemiological research (Coimbra Jr. & Santos, 2000). National surveys remain insufficient to ensure representativeness of Indigenous children. In the most comprehensive nationwide survey, conducted in 2008–2009 across 113 Brazilian Indigenous communities, 25.7% of children under 5 years of age presented stunting (Horta et al., 2013). More recent local studies have reported that approximately 40% of Xavante children (Central Brazil) and over 80% of Yanomami children (Northern Brazil) exhibited linear growth deficits (Ferreira et al., 2016; Orellana et al., 2021).

Although higher rates of stunting among Indigenous children compared to the general population are well documented, there is scarce evidence regarding the occurrence of excess weight. This gap is particularly relevant from a syndemic perspective, which recognizes that shared social and environmental factors contribute to the coexistence of multiple forms of malnutrition (Swinburn et al., 2019). These interacting conditions are likely to disproportionately affect traditional communities, especially in the context of the climate crisis and persistent structural inequalities.

In Northeastern Brazil, Indigenous communities have undergone distinctive processes of cultural reconstruction and political organization after prolonged interactions with non-Indigenous society and a history of ethnic erasure (Arruti, 2013), resulting in complex territorial dynamics. In this region, this study aimed to investigate factors associated with the nutritional status of Indigenous children under 5 years of age. By examining the distribution of height and body mass index (BMI) according to international standards, we aim to provide updated evidence on determinants of early growth and weight status, thereby informing context-sensitive public health strategies to support optimal development among Indigenous children.

## Methods

### Study Design, Location, and Population

This study was a part of the Nutrition, Health, and Food Security Study of Indigenous Peoples in Alagoas (ENSSAIA). ENSSAIA was a cross-sectional household survey conducted between August 2022 and November 2023 with a probabilistic, representative sample of Indigenous peoples of Alagoas. It was developed through a partnership between the Federal University of Alagoas and Indigenous leaders to address gaps in epidemiological data on food and nutrition security in these populations (Dos Santos et al., 2025).

The state of Alagoas is located in the Northeastern region of Brazil, with 3,127,683 inhabitants, of which 20,095 are self-identified Indigenous people. With 1,694,836 Indigenous people (0.83% of the total Brazilian population), the Northeast region has the second largest Indigenous population in the country (31.2%) (IBGE, 2023).

In 2022, 11 Indigenous ethnic groups were identified in 29 communities in Alagoas. Considering the number of individuals registered in the Indigenous Healthcare Subsystem, a sample of 1,113 families was required assuming a 50% prevalence of food insecurity as the primary outcome, a 2.5% sampling error and a 95% confidence level. As described in detail elsewhere (Dos Santos et al., 2025), one community was included per ethnic group; simple random sampling was used for selecting: (i) ethnic groups distributed across more than one community, and (ii) sectors covering one-third of residents in large communities with over 1,500 inhabitants. The final sample included the following Indigenous communities: Aconã, Fazenda Nova Terra, Tingui-Botó, Lajedo do Couro, Baixa do Galo, Roçado, Baixa Fresca, Serra do Engenho, Tanque, Campinhos, Katokinn, Kariri-Xokó, Cocal, and Mata da Cafurna (Dos Santos et al., 2025).

A total of 1,696 households with Indigenous residents in urban and rural areas were registered by the research team with the support of Indigenous community health workers. After exclusion due to refusals (*n* = 73) and unsuccessful interviews (*n* = 223), 1,400 households participated in ENSSAIA (Dos Santos et al., 2025). Of these, 337 households had children under 5 years of age, with a total of 389 individuals. Twins and children without information on date of birth, weight, or height due to incomplete assessments were excluded from the analyses, resulting in an analytical sample of 329 Indigenous children (85% of the total). Children with missing information did not differ significantly from those with complete data with respect to sociodemographic characteristics.

This study followed the guidelines of the 1964 Declaration of Helsinki and its later amendments. All procedures were approved by the Research Ethics Committee of the Federal University of Alagoas and the National Research Ethics Commission (Opinion number: 4.421.186, November 25, 2020). Participation was voluntary after the parents or guardians of the children signed an informed consent form.

### Data Collection and Management

Demographic, socioeconomic, environmental, nutritional, and health information were collected during home visits using semi-structured electronic questionnaires (Epicollect 5, Oxford University, Oxford, United Kingdom). The presence of assets (car, washing machine, computer, DVD player, microwave oven, refrigerator, freezer, motorbike, and clothes dryer) and domestic workers were used to calculate the household wealth index (categorized as below or above the median) using principal component analysis, to capture the relative socioeconomic position of households within the study setting (Filmer & Pritchett, 2001). The first component explained 18.6% of the variation between households and was used to derive weights for each asset that were summed in the wealth index: car (0.4163), washing machine (0.4570), computer (0.4356), DVD player (0.0950), microwave (0.3799), refrigerator (0.2048), freezer (0.3098), motorcycle (0.2639), clothes dryer (0.2030), and maid (−0.1615).

Food insecurity was defined using the 14-question Brazilian Food Insecurity Scale (Fávaro et al., 2007). Households were categorized as food secure (0 points), mild food insecurity (1–5 points), and moderate or severe food insecurity (6–14 points). Maternal work (yes or no), maternal marital status (not living or living with a partner), participation in the Bolsa Família conditional cash transfer program (yes or no), and housing type (masonry or other materials) were assessed. Environmental characteristics included the number of people in the household (≤ 4 or > 4), access to treated water (yes or no), and septic sewage disposal (yes or no). The area of the municipality was categorized as adjacent to rural or urban (IBGE, 2017).

Maternal and perinatal characteristics included maternal age (< 20, 20–35, or > 35 years), parity (primiparous or multiparous), number of prenatal visits (≤ 6 or > 6, based on the Brazilian Ministry of Health recommendation for minimum adequate prenatal care) (Brazil, 2024), preterm birth (gestational age < 37 weeks), type of delivery (vaginal or cesarean section), and birth weight (< 2500, 2500–4000, or > 4000 g, according to information recorded in the child’s health booklet). In addition to the child’s sex and age, recent child health characteristics included morbidity and nutritional factors (hospitalization in the past 12 months; vitamin supplementation in the past 6 months; diarrhea, cough, and fever in the past 2 weeks; yes or no).

To assess nutritional status, an anthropometric examination of the children and their mothers was performed by a trained researcher using standardized techniques (WHO, 1995). Children above 2 years of age and their mothers were weighed using a portable digital scale (Seca, model 813) with a capacity of 200 kg and an accuracy of 100 g. For children under 2 years, the difference between the combined weight of the children and their mothers or guardians alone was used. Measurement of length among children under 2 years was performed using a pediatric stadiometer (Seca, model 417). Standing height of children over 2 years and their mothers was assessed with a portable stadiometer (Seca, model 213). Both length and standing height (hereafter referred to as height) were measured to the nearest 1 mm. BMI was calculated by dividing weight (kg) by the square of height (m).

According to the World Health Organization child growth standards (WHO, 2006), height-for-age (HAZ) and BMI-for-age (BAZ) z-scores were calculated by age and sex using WHO Anthro as an extension of Stata. Stunting was defined as HAZ <−2 z-scores, and overweight as BAZ > + 2 z-scores. Biologically implausible values (± 6 z-scores for HAZ, and ± 5 z-scores for BAZ) were excluded (WHO, 1995). Maternal height was divided into tertiles (1st tertile: ≤ 156, 2nd tertile: > 156 and ≤ 161, and 3rd tertile: > 161 cm) and maternal BMI was classified as underweight (< 18.5 kg/m^2^), normal weight (≥ 18.5 and < 25 kg/m^2^), overweight (≥ 25 and < 30 kg/m^2^), or obesity (≥ 30 kg/m^2^). For adolescent mothers (< 19 years), BAZ was calculated by age and sex using WHO Anthro Plus (de Onis et al., 2007) and categorized as underweight (< −2 z-scores), normal weight (≥ −2 and < + 1 z-score), overweight (> + 1 z-score), or obesity (> + 2 z-scores).

### Data Analysis

The outcomes of interest were HAZ and BAZ (continuous variables, z-scores) in children under 5 years of age. The normal distribution was confirmed using the Shapiro-Wilk test, with a description of means and standard deviations (SD).

The independent variables included socioeconomic, environmental, maternal, perinatal, child health, and nutritional factors. First, the HAZ and BAZ distributions were compared according to the categories of independent variables using the unpaired Student’s t-test for dichotomous variables and ANOVA with Scheffé’s post-hoc test for variables with three or more categories.

Factors associated with anthropometric outcomes were examined using linear regression models, initially adjusting for child age and sex (Model 1), in light of evidence indicating a differential distribution of malnutrition according to these characteristics (Benjamin-Chung et al., 2023). For each outcome, linear regressions were adjusted based on a hierarchical conceptual model defined from a literature review (Victora et al., 2021), with variables at the distal (socioeconomic factors), intermediate (environmental, maternal, and perinatal factors), and proximal (morbidities and nutritional factors) levels, to estimate coefficients (β) and 95% confidence intervals (95%CI). Variables were examined within each level of association and retained in the final models (Model 2) if they were significantly associated with the outcomes. All analyses were performed using Stata 13.0 (Stata Corp., College Station, TX, USA).

## Results

Of the 329 Indigenous children included in the analytic sample, 52% were boys, with a mean (SD) age of 2.4 (1.4) years; 39% were aged 2 years or less. Most children (85.5%) belonged to families benefiting from the Bolsa Família program, and 96% lived in masonry households. Regarding maternal characteristics, the mean maternal age was 28.0 (7.0) years, most mothers reported living with a partner and not working, and 62% had overweight. Among the children, 41.5% were born via cesarean section and 7% had a low birth weight. The occurrence of stunting and overweight was 6.1% and 9.5%, respectively (Table 1).

**Table 1:** Characteristics of Indigenous children under 5 years of age, according to the distribution of height-for-age and body mass index (BMI)-for-age z-scores. Nutrition, Health and Food Security Study of Indigenous Peoples in Alagoas (ENSSAIA)

	Anthropometric indices^a^						
	Height-for-age				BMI-for-age		
	*N*^b^ (%)	Mean (SD)	*p*		*N*^b^ (%)	Mean (SD)	*p*
***Total***	329 (100.0)	−0.38 (1.09)		316 (100.0)		0.50 (1.10)	
** Child sex**			0.13				0.05
Male	171 (52.0)	−0.47 (1.10)			165 (52.2)	0.61 (1.21)	
Female	158 (48.0)	−0.29 (1.08)			151 (47.8)	0.37 (0.97)	
** Child age**			0.52				0.01
≤ 2 years	125 (38.0)	−0.33 (1.19)			120 (38.0)	0.68 (1.10)	
> 2 years	204 (62.0)	−0.41 (1.03)			196 (62.0)	0.38 (1.09)	
***Socioeconomic characteristics***							
** Household wealth index**			0.02				0.91
Below the median	160 (48.8)	−0.53 (1.07)			155 (49.9)	0.49 (1.09)	
Above the median	168 (51.2)	−0.25 (1.10)			160 (50.1)	0.50 (1.13)	
** Household food insecurity** ^**c**^			< 0.01				1.00
Food secure	83 (25.3)	0.00 (1.17)			77 (24.4)	0.50 (1.14)	
Mild food insecurity	163 (49.7)	−0.46 (1.00)			159 (50.5)	0.50 (1.06)	
Moderate or severe food insecurity	82 (25.0)	−0.64 (1.09)			79 (25.1)	0.50 (1.18)	
** Maternal work**			0.51				0.26
No	266 (83.9)	−0.37 (1.09)			255 (83.6)	0.47 (1.12)	
Yes	51 (16.1)	−0.26 (1.10)			50 (16.4)	0.67 (1.07)	
** Maternal marital status**			0.85				0.93
Not living with a partner	74 (23.4)	−0.33 (0.94)			74 (24.3)	0.49 (1.13)	
Living with a partner	243 (76.6)	−0.36 (1.14)			231 (75.7)	0.51 (1.11)	
** Cash transfer program**			0.58				0.21
No	47 (14.5)	−0.30 (1.36)			44 (14.0)	0.69 (1.03)	
Yes	278 (85.5)	−0.39 (1.05)			268 (86.0)	0.46 (1.12)	
** Housing type** ^**e**^			< 0.01				0.80
Masonry	315 (96.0)	−0.35 (1.06)			303 (96.2)	0.50 (1.12)	
Other materials	13 (4.0)	−1.27 (1.42)			12 (3.8)	0.42 (0.73)	
***Environmental characteristics***							
** Municipal area** ^**d**^			0.84				0.96
Adjacent rural	237 (72.0)	−0.37 (1.13)			224 (70.9)	0.50 (1.12)	
Urban	92 (28.0)	−0.40 (1.00)			92 (29.1)	0.49 (1.08)	
** Number of residents in the household**			0.16				0.40
≤ 4 people	190 (57.7)	−0.31 (1.03)			183 (57.9)	0.45 (1.12)	
> 4 people	139 (42.3)	−0.48 (1.17)			133 (42.1)	0.56 (1.08)	
** Access to treated water**			0.69				0.28
No	113 (34.5)	−0.35 (1.11)			109 (34.6)	0.40 (1.15)	
Yes	215 (65.5)	−0.40 (1.09)			206 (65.4)	0.55 (1.08)	
** Septic waste disposal**			0.25				0.73
No	125 (37.7)	−0.30 (1.08)			118 (37.5)	0.47 (1.07)	
Yes	206 (65.5)	−0.44 (1.10)			197 (62.5)	0.51 (1.13)	
***Maternal characteristics***							
** Maternal height (cm)**			< 0.01				0.38
1st tertile (≤ 156)	106 (34.7)	−0.68 (0.94)			102 (35.2)	0.48 (1.03)	
2nd tertile (> 156 to ≤ 161)	106 (35.0)	−0.36 (1.19)			102 (35.2)	0.45 (1.12)	
3rd tertile (> 161)	90 (30.3)	0.04 (1.06)			86 (29.6)	0.66 (1.18)	
** Maternal nutritional status** ^**f**^			0.44				0.02
Underweight	5 (1.7)	−1.14 (1.28)			5 (1.8)	−0.29 (1.20)	
Normal weight	106 (36.3)	−0.33 (1.10)			106 (36.5)	0.38 (1.00)	
Overweight	108 (37.0)	−0.37 (1.15)			108 (37.2)	0.50 (1.19)	
Obesity	73 (25.0)	−0.33 (1.00)			71 (24.5)	0.82 (1.07)	
** Maternal age (years)**			0.08				0.29
< 20	33 (10.0)	−0.58 (1.27)			32 (10.2)	0.22 (0.90)	
20 to 35	247 (75.1)	−0.42 (1.07)			238 (75.3)	0.54 (1.15)	
> 35	49 (14.9)	−0.08 (1.04)			46 (14.5)	0.46 (1.00)	
** Parity**			0.34				0.38
Primiparous	102 (32.6)	−0.26 (1.06)			100 (33.3)	0.41 (1.06)	
Multiparous	211 (67.4)	−0.39 (1.10)			201 (66.7)	0.53 (1.13)	
***Perinatal characteristics***							
** Number of prenatal visits**			0.56				0.18
≤ 6	58 (20.0)	−0.40 (1.18)			55 (19.8)	0.32 (1.23)	
> 6	232 (80.0)	−0.31 (1.06)			223 (80.2)	0.54 (1.09)	
** Prematurity** ^**g**^			0.44				0.64
No	307 (94.2)	−0.37 (1.11)			295 (93.9)	0.49 (1.08)	
Yes	19 (5.8)	−0.57 (0.90)			19 (6.1)	0.61 (1.43)	
** Type of delivery**			0.02				< 0.01
Vaginal	190 (58.4)	−0.50 (1.08)			183 (58.6)	0.31 (1.02)	
Cesarean section	135 (41.6)	−0.22 (1.11)			129 (41.4)	0.77 (1.16)	
** Birth weight (g)**			0.02				< 0.01
< 2500	21 (7.0)	−0.92 (0.86)			20 (6.9)	0.11 (1.07)	
* ≥* 2500 to ≤ 4000	255(85.0)	−0.31 (1.11)			247 (85.2)	0.50 (1.12)	
> 4000	24 (8.0)	−0.05 (1.05)			23 (7.9)	1.14 (1.10)	
***Morbidities and nutritional factors***							
** Hospitalization** ^**h**^			0.39				0.96
No	309 (94.5)	−0.37 (1.10)			296 (94.3)	0.49 (1.09)	
Yes	18 (5.5)	−0.60 (0.98)			18 (5.7)	0.50 (1.36)	
** Vitamin A supplementation** ^**i**^			0.33				0.09
No	59 (20.0)	−0.52 (1.07)			51 (18.2)	0.28 (1.11)	
Yes	233 (80.0)	−0.37 (1.09)			229 (81.8)	0.56 (1.06)	
** Diarrhea** ^**j**^			0.93				0.73
No	284 (86.8)	−0.38 (1.07)			272 (86.6)	0.48 (1.12)	
Yes	43 (13.2)	−0.40 (1.25)			42 (13.4)	0.55 (0.98)	
** Cough and fever** ^**j**^			0.52				0.08
No	163 (49.8)	−0.43 (1.18)			160 (50.9)	0.60 (1.15)	
Yes	164 (50.2)	−0.35 (1.01)			154 (49.1)	0.38 (1.04)	

^a^Height-for-age and BMI-for-age z-scores calculated according to the WHO growth standards

^b^Totals may differ due to missing information on height or weight or exclusion of biologically implausible HAZ or BAZ values

^c^Measured by the Brazilian Food Insecurity Scale and referring to the past 3 months

^d^Municipal area: classification of municipalities by Rural-Urban typology according to IBGE

^e^Housing type: other materials (rammed earth, wood)

^f^Maternal BMI classified as underweight (< 18.5 kg/m^2^), normal weight (≥ 18.5 and < 25 kg/m^2^), overweight (≥ 25 and < 30 kg/m^2^), or obesity (≥ 30 kg/m^2^) among adult mothers. For adolescent mothers (< 19 years), BAZ was categorized as underweight (< −2 z-scores), normal weight (≥ −2 and < + 1 z-score), overweight (> + 1 z-score), or obesity (> + 2 z-scores)

^g^Prematurity: gestational age < 37 weeks

^h^Hospitalization: in the past year

^i^Vitamin A supplementation: in the past 6 months

^j^Diarrhea, cough and fever: in the past 2 weeks

In models controlling for the child’s age and sex (Model 1), socioeconomic and environmental characteristics were associated with HAZ, but not with BAZ (Tables 2 and 3). For HAZ, positive associations with wealth index and masonry households were observed, while food insecurity was related to lower HAZ. Maternal nutritional status, cesarean delivery, and birth weight were associated with both anthropometric outcomes.

**Table 2:** Factors associated with height-for-age z-score among Indigenous children under 5 years of age. Nutrition, Health and Food Security Study of Indigenous Peoples in Alagoas (ENSSAIA)

		Height for age z-score				
		Model 1^a^		Model 2^b^		
		ß	95%CI	ß	95%CI	*P*
***Socioeconomic characteristics***						
** Household wealth index**						
Below the median		Ref.				
Above the median		0.25	0.02, 0.49			
** Household food insecurity**						
Food secure		Ref.		Ref.		
Mild food insecurity		−0.44	−0.73, −0.16	−0.36	−0.64, −0.08	0.01
Moderate or severe food insecurity		−0.61	−0.94, −0.27	−0.37	−0.70, −0.03	0.03
** Housing type**						
Other materials		Ref.		Ref.		
Masonry		0.93	0.33, 1.53	0.71	0.10, 1.32	0.02
***Environmental characteristics***						
** Number of residents in the household**						
≤ 4 people		Ref.				
> 4 people		−0.17	−0.41, 0.06			
***Maternal characteristics***						
** Maternal height (cm)**						
1st tertile (≤ 156)		Ref.		Ref.		
2nd tertile (> 156 to ≤ 161)		0.31	0.02, 0.60	0.25	−0.03, 0.53	0.08
3rd tertile (> 161)		0.71	0.41, 1.01	0.54	0.24, 0.85	< 0.01
** Maternal age (years)**						
< 20		Ref.				
20 to 35		0.17	−0.23, 0.57			
> 35		0.53	0.04, 1.01			
***Perinatal characteristics***						
** Type of delivery**						
Vaginal		Ref.				
Cesarean section		0.27	0.03, 0.51			
** Birth weight (g)**						
< 2500		−0.60	−1.08, −0.12	−0.52	−0.99, −0.06	0.02
* ≥* 2500 to ≤ 4000		Ref.		Ref.		
> 4000		0.29	−0.16, 0.74	0.11	−0.33, 0.55	0.62

^a^Model 1: linear regression models for each variable of interest, adjusting for child age and sex

^b^Model 2: multiple linear regression model adjusting for child age and sex, and all variables retained at each level of association according to a hierarchical conceptual framework for factors associated with the outcome

**Table 3:** Factors associated with body mass index (BMI)-for-age z-score among Indigenous children under 5 years of age. Nutrition, Health and Food Security Study of Indigenous Peoples in Alagoas (ENSSAIA)

	BMI-for-age z-score				
	Model 1^a^		Model 2^b^		
	***ß***	95%CI	***ß***	95%CI	P
***Socioeconomic characteristics***					
** Bolsa Familia cash transfer program**					
No	Ref.				
Yes	−0.15	−0.50, 0.20			
***Maternal characteristics***					
** Maternal nutritional status**					
Normal weight	Ref.		Ref.		
Underweight	−0.96	−1.94, 0.11	−1.02	−2.00, −0.03	0.04
Overweight	0.11	−0.17, 0.40	0.04	−0.24, 0.32	0.77
Obesity	0.48	0.16, 0.81	0.32	−0.01, 0.65	0.06
***Perinatal characteristics***					
** Number of prenatal visits**					
≤ 6	Ref.				
> 6	0.25	−0.06, 0.58			
** Type of delivery**					
Vaginal	Ref.		Ref.		
Cesarean section	0.46	0.22, 0.70	0.38	0.14, 0.63	< 0.01
** Birth weight (g)**					
< 2500	−0.34	−0.83, 0.15	−0.24	−0.73, 0.24	0.32
* ≥* 2500 to ≤ 4000	Ref.		Ref.		
> 4000	0.63	0.17, 1.10	0.44	−0.02, 0.90	0.06

^a^Model 1: linear regression models for each variable of interest, adjusting for child age and sex

^b^Model 2: multiple linear regression model adjusting for child age and sex, and all variables retained at each level of association according to a hierarchical conceptual framework for factors associated with the outcome

In the adjusted model for HAZ (Model 2, Table 2), among socioeconomic factors, Indigenous children from households with moderate or severe food insecurity exhibited a −0.37 z lower HAZ than those in food-secure households (95%CI: −0.70, −0.03). Even though only 4% of Indigenous children were living in other housing types, masonry households were associated with a + 0.71 z higher HAZ (95%CI: 0.10, 1.32). Among maternal and perinatal characteristics, maternal height was positively associated with HAZ (3rd tertile: 0.54 z; 95%CI: 0.24, 0.85, compared with the 1st tertile). Additionally, a low birth weight was associated with a −0.52 z lower HAZ than an adequate birth weight (95%CI: −0.99, −0.06).

Socioeconomic and environmental characteristics were not significantly associated with child BAZ in the adjusted model (Model 2, Table 3). Compared with Indigenous children born to mothers with adequate weight, those who were born to mothers with underweight had a −1.02 z lower BAZ (95%CI: −2.00, −0.03). Cesarean section was associated with a higher BAZ (β: 0.38 z; 95%CI: 0.14, 0.63). Indigenous children who were born weighing more than 4000 g had a significantly higher BAZ than their counterparts with low birth weight (β: 0.69 z; 95%CI: 0.04, 1.34; data not shown in tables).

## Discussion

This study characterized the current nutritional status of Indigenous children under 5 years of age from communities in Northeastern Brazil, where overweight (9.5%) was more frequently observed than stunting (6.1%). While the main factors associated with HAZ included socioeconomic characteristics, maternal height, and birth weight, BAZ was mainly influenced by maternal nutritional status and cesarean section.

According to the Brazilian National Study of Child Food and Nutrition (ENANI-2019), a representative household survey of children under 5 years of age in the general population, the prevalence of stunting was 7.0%, with a mean HAZ of −0.30 z (Castro et al. 2023a). However, this nationwide survey did not systematically include Indigenous populations. Using a cross-sectional population-based approach, the 2008–2009 National Survey of Indigenous Health and Nutrition among children under 5 years of age reported a stunting prevalence of 13.9% in the Northeastern region of the country (Horta et al., 2013). This estimate is higher than that observed in ENSSAIA. Regarding BAZ, overweight was observed in 10.1% of Brazilian children in the general population (Castro et al. 2023b). Comparable estimates for BAZ among Indigenous children were not available in the 2008–2009 National Survey of Indigenous Health and Nutrition.

The similarity in the prevalence of stunting and overweight between ENANI-2019 and ENSSAIA represents a novel finding in studies involving Indigenous populations, highlighting the ongoing progression of the nutrition transition (Castro et al. 2023b). This pattern is relevant in light of the processes of ethnic reemergence observed in recent decades in Northeastern Brazil (Arruti, 2013), and underscores the diversity of contexts across Indigenous communities. Amid historical discrimination and greater food insecurity observed in the study setting, the protection of Indigenous territories should be pivotal to revitalize traditional food systems and health practices.

In this sense, Demographic and Health Surveys in low- and middle-income countries have shown that declines in mean HAZ at 0–36 months were accompanied by declines in values at both the 5th and 95th percentiles of the distribution. Thus, suboptimal growth patterns in the first years of life may be considered a population condition, with evidence indicating that children across the entire HAZ distribution experience declines in nutritional status adequacy (Roth et al., 2017). Examining factors associated with anthropometric indices, in addition to classifying forms of malnutrition using standard cut-offs, is particularly important in populations affected by social and health inequalities.

Socioeconomic characteristics are crucial for child growth and influence HAZ up to 5 years of age. In this study, a lower mean HAZ was observed among children in more disadvantaged socioeconomic conditions, whereas this pattern was not confirmed for BAZ. A systematic review of studies from 2012 to 2022 examined the association between household food insecurity and stunting in children under 5 years of age across 17 countries, predominantly low-middle or low-income countries. Children living in food-insecure households were more likely to have stunting, with odds ratios (ORs) ranging from 1.13 to 6.70 (Lye et al., 2023).

It is noteworthy that 25% of the study population reported moderate or severe food insecurity, a proportion similar to that found in Latin America (27.2%), but substantially higher than recent estimates for Brazil (9.4%) (FAO, 2024; IBGE, 2024). Severe food insecurity is characterized by the experience of hunger, whereas moderate food insecurity reflects compromised food quantity and quality. Adequate and sufficient food is a fundamental human right and is essential to meet children’s growth requirements, being particularly relevant for HAZ among Indigenous children in the present analysis.

Additional evidence supports the importance of socioeconomic factors for optimal child growth. In Brazil, stunting declined by approximately 50% between 1996 and 2007. Of this reduction, 11.1% was attributed to increased family purchasing power, 13.2% to higher maternal education, 5.9% to expanded access to healthcare, and 2.2% to improvements in sanitation (Monteiro et al., 2009). Conditional cash transfer programs such as Bolsa Família support low-income families and have played an important role in enhancing socioeconomic conditions. As 85.5% of children in this study were beneficiaries of the program, this widespread coverage may be relevant to the distribution of HAZ in the region. A systematic review of studies between 2016 and 2022, most of which were conducted in low- and middle-income countries (81.8%), found that cash transfer programs were associated with a reduction in malnutrition as measured by HAZ (Lisboa et al., 2023).

Regarding maternal nutritional status, this study confirmed positive associations between maternal height and BMI and children’s HAZ and BAZ, respectively. Data from 239 Demographic and Health Surveys (1990–2021) showed that children born to mothers with shorter stature were 136% more likely to have stunting (OR: 2.36; 95%CI: 2.24, 2.47) compared with those with taller mothers (Büttner et al., 2023). Pooled analyses of cohort studies conducted in low- and middle-income countries between 1987 and 2014 further suggested that each 1 cm increase in maternal height was associated with a 0.5 to 0.7 cm increase in child length in the first 2 years of life (Mertens et al., 2023). Similarly, mothers with a BMI < 18.5 kg/m^2^ were 90% more likely to have children with underweight (OR: 1.90; 95%CI: 1.83, 1.97) compared with their counterparts (Büttner et al., 2023). Among Brazilian children under 5 years of age, maternal overweight was positively associated with child overweight, although these data were not representative of Indigenous households in the country (Farias et al., 2023). These results illustrate a cycle in which mothers affected by malnutrition are more likely to have children who also suffer from various forms of malnutrition (Büttner et al., 2023; Farias et al., 2023), highlighting the role of intergenerational dynamics of poverty and shared household environment, as well as the impact of maternal malnutrition since pregnancy.

Among the Indigenous children in this study, a higher BAZ was associated with cesarean delivery, which was observed in 41.6% of the participants. A systematic review and meta-analysis of nine cohort studies published between 2019 and 2020 found that cesarean delivery was associated with the occurrence of obesity in preschool children (relative risk: 1.12; 95%CI: 1.02, 1.22) (Zhang et al., 2022). Studies suggest that the lack of exposure to maternal microbiota in cesarean delivery may be a potential biological mechanism for the development of obesity (Masukume et al., 2019; Neu & Rushing, 2011). However, factors such as high pre-pregnancy BMI, increased gestational weight gain, and larger infant size should be carefully considered as these characteristics are likely associated with cesarean delivery and contribute to a higher BAZ in children (Quecke et al., 2022).

Birth weight was also found to be an important factor influencing HAZ and BAZ in Indigenous children under 5 years of age. A study based on data from the Demographic and Health Surveys of low- and middle-income countries conducted between 2000 and 2015 also linked low birth weight to growth faltering. Children who experience both low birth weight and shorter length are at increased risk of mortality, and growth deficits are exacerbated in the postnatal period, particularly in the first 2 years of life (Victora et al., 2021). In contrast, meta-analyzed data showed that high birth weight was associated with a significant increase in the odds of obesity in children and adolescents aged between 2 and 18 years (OR: 2.07; 95%CI: 1.91, 2.24) (Yu et al., 2011). In a study of 614 children living in urban areas in the Brazilian Amazon region, positive and significant tracking of the adequacy of nutritional status was observed at a considerable magnitude from birth to 5 years of age, for both linear growth and weight gain (Lourenço et al., 2023). Considering that the incidence of stunting is highest between birth and the first 6 months of life in low- and middle-income regions (Benjamin-Chung et al., 2023), it is essential to integrate the influences of fetal growth conditions in order to optimize postnatal growth processes.

This study has several limitations. First, while the findings are not representative of the entire Indigenous Brazilian population, they offer valuable insights into malnutrition dynamics among Indigenous children in Northeastern Brazil, amid relevant processes of ethnic reemergence. Second, the cross-sectional design limits interpretations on causal relationships. Third, logistical constraints prevented the collection of data on household shared environment and food intake, restricting the analysis of these associations. Nonetheless, this study’s strengths include standardized anthropometric assessment procedures, thorough training of the field team, and the active involvement and agreement of Indigenous community leaders. These factors enhance the reliability of the findings and support future research and data updates in Indigenous health.

In conclusion, the nutritional status of Indigenous children under 5 years of age in Northeastern Brazil reflects a challenging scenario, as suboptimal growth persists alongside a notable prevalence of overweight. Socioeconomic characteristics were particularly associated with HAZ, whereas maternal nutritional status and perinatal characteristics influenced both height and weight status. These findings should be interpreted within a broader context of structural inequities affecting Indigenous peoples in Brazil, as well as the relevance of strengthening traditional food and health practices within Indigenous territories, thereby contributing to improved nutritional outcomes in children.

## Data Availability

The datasets used in this study are available from the corresponding author upon reasonable request.

## Code Availability

Code is available from the corresponding author upon reasonable request.
